# *In vitro* Non-Small Cell Lung Cancer Inhibitory Effect by New Diphenylethane Isolated From Stems and Leaves of *Dioscorea oppositifolia* L. via ERβ-STAT3 Pathway

**DOI:** 10.3389/fphar.2021.622681

**Published:** 2021-02-02

**Authors:** Mengnan Zeng, Yingjie Ren, Beibei Zhang, Shengchao Wang, Meng Liu, Jufang Jia, Pengli Guo, Qinqin Zhang, Xiaoke Zheng, Weisheng Feng

**Affiliations:** ^1^ College of Pharmacy, Henan University of Chinese Medicine, Zhengzhou, China; ^2^ The Engineering and Technology Center for Chinese Medicine Development of Henan Province, Zhengzhou, China; ^3^ Henan University of Chinese Medicine, Co-construction of Collaborative Innovation Center for Chinese Medicine and Respiratory Diseases by Henan & Education Ministry of P.R. China, Zhengzhou, China

**Keywords:** new diphenylethane, non-small cell lung cancer, ERβ, stat3, stems and leaves of *Dioscorea oppositifolia* L

## Abstract

Lung cancer is the most leading cause of cancer mortality throughout the world, of which about 85% cases comprise the non-small cell lung cancer (NSCLC). Estrogen and estrogen receptors are known to be involved in the pathogenesis and development of lung cancer. *Dioscorea oppositifolia* L. is a traditional Chinese medicine and a nutritious food, and can be an excellent candidate as an anti-cancer agent owing to its estrogen-like effects. However, the stems and leaves of *D. oppositifolia* L. are piled up in the field as a waste, causing environmental pollution and waste of resources. In the present study, a new diphenylethane (D1) was isolated from the stems and leaves of *D. oppositifolia* L. It was observed that D1 reduced the cell viability, migration, energy metabolism, and induced apoptosis in the A549 cells. Mechanistic studies showed that D1 reduced the STAT3 nuclear localization and downregulated the expression of the STAT3 target genes like Mcl-1, Bcl-xL and MMP-2 that are involved in the cell survival and mobility. Moreover, our results indicated that D1 exhibited estrogenic activities mediated by ER*β*, and antagonising ER*β* decreased the cytotoxic effect of D1 in A549 cells. In addition, inhibition of the nuclear translocation of STAT3 did not interfere with the binding of D1 and ER*β*. However, after antagonizing ER*β*, the nuclear translocation of STAT3 increased, thereby demonstrating that STAT3 was the downstream signaling molecule of ER*β*. In conclusion, the D1 mediated anti-NSCLC *in vitro* effects or at least in part can be attributed to the ER*β*-STAT3 signaling. Our findings suggest the role of D1 in treating NSCLC on a molecular level, and can help to improve the comprehensive utilization rate of *D. oppositifolia* L.

## Introduction

According to the Global Annual Cancer Report of 2019, lung cancer remains the most malignant tumor, in terms of both morbidity (11.6%) and mortality (18.4%). Among the different variants, the non-small cell lung cancer (NSCLC) accounts for approximately 85% of the lung cancer incidences (Cong et al., 2019; Siegel et al., 2020). At present, a significant progress has been made in the treatment of NSCLC. Targeted therapy and immunotherapy have significantly increased the 5-years survival rate (Herbst et al., 2018). However, with the emergence of new problems, such as high recurrence rate, metastasis and drug resistance, there is an urgent need of new treatment options that are highly efficient, low-cost and with weak side-effects. Some traditional Chinese medicines have certain anti-tumor effects, which imply to be detoxifying, heat-clearing and strengthening the foundation of body, according to the traditional Chinese medicine (Wang et al., 2019). These medicines typically have multiple targets, functioning at multiple stages and showing diverse effects. Furthermore, they have low toxic and side effects, improve the body’s immunity, and are not prone to drug resistance. Therefore, the anti-tumour traditional Chinese medicines have become a popular area of research. With the gradual understanding of the traditional Chinese medicine, it has been realised that the active anti-tumour ingredients (e.g., baicalein, matrine and oridonin) extracted from the traditional Chinese medicine are suitable for multi-target therapy (Han et al., 2016).

*Dioscorea oppositifolia* L. is documented in the “Shennong Bencaojing” and is regarded as a model of the “chemical similarity of medicine and food” principle in ancient China, besides promoting the functions of lungs and kidneys (Ren et al., 2019a). Modern pharmacological research shows that it has the biological activities of lowering blood sugar and blood pressure, as well as the anti-inflammatory and anti-tumour effects (Hsu et al., 2014; Ma et al., 2017; Ren et al., 2019b). However, during the collecting process of *D. oppositifolia* L., its aerial parts (stems and leaves) are piled up in the field as waste, causing environmental pollution and a waste of resource. At present, the non-medicinal parts of many traditional Chinese medicine plants or their extracts have been used in clinical practice, such as *Eucommia ulmoides*, a cheap and common plant which has anti-hypertensive effects (Hu et al., 2020). Chlorogenic acid extracted from the *E. ulmoides* leaves is known to exhibit anti-bacterial and anti-inflammatory effects (Zhao et al., 2020). Earlier, we studied the chemical constituents in the stems and leaves of *D. oppositifolia* L., and isolated sesquiterpenoids, diphenylethanes and other compounds (Ren et al., 2019a; Ren et al., 2019b). We found that these metabolites exhibit estrogen-like activities, which is mediated by the estrogen receptor (ER). Several studies have shown that estrogen therapy can improve the prognosis of NSCLC patients and improve the efficacy, and has been gradually used in the treatment of NSCLC (Kawai, 2014; Samuel et al., 2019; Abdulaziz et al., 2020). STAT3 is known to regulate the proliferation, differentiation and migration of lung cancer cells by activating the genes associated with the cell cycle progression, inducing survival genes and genes playing role in the invasion and metastasis (Xiong et al., 2020). In the present study, a new diphenylethane compound was isolated from the stems and leaves of *D. oppositifolia* L., and its intervention on A549 cells was studied from the perspective of ER and STAT3. Our study provides important information for exploring the pharmacological actions of the stems and leaves of *D. oppositifolia* L., and in improving the comprehensive utilization rate of *D. oppositifolia* L. In addition, this study also provides a theoretical basis for the discovery of new drugs in the treatment of NSCLC.

## Materials and Methods

### General Experimental Procedures

NMR spectra (500 MHz for ^1^H NMR and 125 MHz for ^13^C NMR) were measured on a Bruker Avance Ⅲ 500 spectrometer, using tetramethylsilane as the internal standard (Bruker, Germany). Mass spectra were recorded on a Bruker maXis HD mass spectrometer (Bruker, Germany). UV spectra were measured using a Thermo EVO 300 spectrometer (Thermo, United States). IR spectra was recorded on a Thermo Nicolet IS 10 spectrometer (Thermo, United States). Analytical HPLC was performed using a Waters Alliance 2,695 system (Waters, Milford, MA, United States) equipped with a Waters 2998 DAD detector and a Platisil ODS C18 column (4.6 × 250 mm, 5 μm, Dikma, Beijing, China). A Beijing Chuangxintongheng Science LC 3000 HPLC system (Chuangxintongheng, Beijing, China) was used for semi-preparative high performance liquid chromatography (HPLC), equipped with P3000 pumps and an UV/VIS 3000 detector. HPLC separations were performed using a YMC-Pack ODS-A column (20 × 250 mm, 5 μm; YMC, Kyoto, Japan) with a Diaion HP-20 (Mitsubishi Chemical Corporation, Tokyo, Japan), Toyopearl HW-40C, MCI CHP-20 (TOSOH Corp., Tokyo, Japan), and Sephadex LH-20 (40–70 μm, Amersham Pharmacia Biotech AB, Uppsala, Sweden). TLC was performed using glass precoated silica gel GF254 plates (10–40 m, Marine Chemical Industry, Qingdao, China). Silica gel (200–300 mesh, Marine Chemical Industry, Qingdao, China) was used for column chromatography (CC) (Ren et al., 2019b).

### Plant Material

The stems and leaves of *D. oppositifolia* L. were collected in September 2017 in Wenxian (112°51′39″ to 113°13′20″ east longitude, 34°52′ to 35°2′48″ north latitude), Jiaozuo city, Henan province, People’s Republic of China, and was identified by Prof. Sui-qing Chen of Henan University of Chinese Medicine. A voucher specimen (No. 20171115A) was stored in the Department of Natural Medicinal Chemistry, School of Pharmacy, Henan University of Chinese Medicine, Zhengzhou, China (Ren et al., 2019b).

### Extraction and Isolation

Dried stems and leaves (40 kg) were extracted with aqueous acetone (50% v/v) twice (2 × 100 L, 10 min each time) at room temperature. The extracts (5.8 kg) were suspended in water (8 L) and then partitioned successively with petroleum ether (5 × 8 L) and dichloromethane (5 × 8 L). The dichloromethane extract (29.3 g) was separated using silica gel column chromatography (CC) with a gradient system of petroleum ether–ethyl acetate (100:0–0:100) to produce five fractions (Fr. 1–Fr. 5). Fr. Four was applied to silica gel CC and eluted with ethyl acetate–methanol (100:1–0:1) to generate three fractions (Fr. 4-1–Fr. 4-3). Fr. 4-1 was fractionated using silica gel CC and eluted with dichloromethane–methanol (100:1–0:1) to generate five fractions (Fr. 4-1-1–Fr. 4-1-5). Fr. 4-1-4 was subjected to silica gel CC and eluted with petroleum ether–ethyl acetate (4:1–0:1) and ethyl acetate–methanol (20:1–0:1) to generate five fractions (Fr. 4-1-4-1–Fr. 4-1-4-5). Fr. 4-1-4-4 was subjected to silica gel CC and eluted with petroleum ether–ethyl acetate (16:1–0:1) and ethyl acetate–methanol (10:1–0:1) to generate five fractions (Fr. 4-1-4-4-1–Fr. 4-1-4-4-5). Fr. 4-1-4-4-2 was separated on a Toyopearl HW-40 column (MeOH) and further purified by semi-preparative HPLC [(MeOH:H_2_O, 57:43 v/v) and (MeOH:H_2_O, 57:43 v/v)] to yield the compound D1 (5.52 mg, t_*R*_ = 17.1 min) (Ren et al., 2019b).

### Cell Culture and Treatment

The human lung adenocarcinoma cells A549 and human breast cancer cells MCF-7 were obtained from the American Type Culture Collection (Manassas, VA, United States). The A549 and MCF-7 cells were cultured in Dulbecco’s modified Eagle’s medium (DMEM) supplemented with 2 mM L-glutamine, 50 units/ml penicillin, 50 μg/ml streptomycin, and 10% heat-inactivated foetal bovine serum (FBS) at 37°C under a humidified atmosphere of 5% CO_2_ in air. Prior to the experiments involving the treatment with estrogen, MCF-7 cells were cultured in a hormone-free medium (phenol red-free DMEM with 10% v/v charcoal-stripped FBS) for 7 days (Ren et al., 2019a)

### Cell Viability

A549 cells were seeded in 96-well plates in DMEM with 10% v/v FBS. The density was 5,000 cells/well, and cells were divided into the Control group, D1 (25 μM and 50 μM) group, and D1 (25 μM and 50 μM) + STAT3 inhibitor (AG490, 10 μM) group. After 24 or 48 h, the MTT assay was used to detect cell viability. The cell viability was proportional to absorbance.

MCF-7 cells were seeded on a 96-well plates in phenol red-free DMEM with 10% v/v charcoal-stripped FBS, cells were divided into the control group, groups of D1 (25, 50 μM); in another set of experiments, the specific ER*β* antagonist (THC, 1 μM) was added 30 min before D1 to evaluate, whether the observed effects elicited by D1 were mediated by ER*β* (Zeng et al., 2018). After 24 h, the MTT assay was used to detect cell viability.

### Apoptosis

A549 cells were seeded on 6-well plates (4× 10^5^ cells/well) and allowed to adhere overnight. Cells were divided into the Control group, D1 (25 μM and 50 μM) group, D1 (25 μM and 50 μM) + AG490 (10 μM) group, D1 (25 μM and 50 μM) + THC (1 μM) group. 24 h later, the po-apoptotic effects of D1 on A549 cells were evaluated using the 7-AAD/PE double staining according to the manufacturer’s instructions (BD Biosciences 556547, United States). Flow cytometry was performed using a FACS AriaIII (BD Biosciences, United States) utilizing 5,000 events. Three independent experiments were performed (Zeng et al., 2020).

### Cell Migration

Assay for cell migration was performed using a 96-well plate (E190236X, PerkinElmer, United States) and 16-well plate (CIM-Plate16, ACEA, Belgium). Cells (2000 cells/well) were divided into the Control group, D1 (25 μM and 50 μM) group, D1 (25 μM and 50 μM) + AG490 (10 μM) group, D1 (25 μM and 50 μM) + THC (1 μM) group, D1 (25 μM and 50 μM) + specific estrogen receptor β agonist (PPT, 1 μM) group in 96-well plates, the movement of A549 cells was monitored in real time by high-content imaging system (Opera Phenix, PerkinElmer, United States). The displacement was normalized against the Harmony 4.8. In another experiment, 160 μl medium was added to the lower chamber of the CIM-Plate16, the upper and lower chambers were assembled, and 30 μl complete medium was added to the upper chamber. Subsequently, 100 μl the cell suspension (group as described above) was added to the chamber of the CIM-Plate16, xCELLigence system for real-time analysis of cell migration. The results were expressed by the cell migration kinetic curve (time-versus-index).

### OCR and ECAR

Oxygen consumption rate (OCR) and extracellular acidification rate (ECAR) were measured using a Seahorse analyzer (Agilent Technologies, United States) and a Seahorse XF Cell Mito Stress Test Kit (Agilent Technologies, United States). Cells were divided into Control group and D1 (25 μM and 50 μM) group. Before the test, the culture medium was replaced with a specific medium (Seahorse XF DMEM, pH 7.4), to which oligomycin (1 mol/L), FCCP (2 mol/L), and rotenone/antimycin A (0.5 mol/L) were added. After detection, the data were normalized through the cell count, and then the OCR value per 10,000 cells was calculated. In addition, glucose (10 mM), oligomycin (2 μM), and 2-DG (50 mM) were diluted in assay medium and injected into ports A, B, and C, respectively, of the Seahorse analyzer. The assay was performed using the glycolytic stress test assay protocol, and the ECAR value per 10,000 cells was calculated (Jung et al., 2016).

### Cellular Immunofluorescence

Assay for cellular immunofluorescence was performed in 96-well plates (E190236X, PerkinElmer, United States). After treatment, the A549 cells were divided into the Control group, D1 (25 μM and 50 μM) group, D1 (25 μM and 50 μM) + THC (1 μM) group, D1 (25 μM and 50 μM) + PPT (1 μM) group, and the MCF-7 cells were divided into the Control group, D1 (25 μM and 50 μM) group, the cells were fixed with 4% paraformaldehyde for 20 min at room temperature. The cell monolayers were blocked for 90 min and then incubated with primary antibodies (ER*β* ab3576 Abcom, STAT3 9139S CST, p-STAT3 9145S CST, Bcl-XL ab32370 Abcam, MMP-2 ab92536 Abcam, MCL-1 ab32087 Abcam), diluted in blocking buffer (1:200, 50 μl) overnight at 4°C. After washing with PBS with Tween-20 (PBST) buffer, the cell layers were stained with the anti-mouse IgG or anti-rabbit IgG (1918277 or 1981155, 1:500, 50 μL; ThermoFisher Scientific, United States) for 1 h at room temperature, rinsed, and scanned using an High-content imaging system (Opera Phenix, PerkinElmer, United States). The relative protein expression level was normalized against the Harmony 4.8 (Zeng et al., 2020).

### Molecular Docking

The ER*β* protein was used as the receptor (PDB: 1U3Q), and D1 was employed as a ligand. Sphere ball (active pocket) was defined according to the ligand. Molecular docking was performed using the Discovery Studio Client V3.0 (Accelrys software Inc.).

### Statistical Analysis

Data were analyzed using the SPSS software 20.0 (IBM, United States). Statistical significance was assessed in comparison with the respective control for each experiment using one-way analysis of variance. *p* values less than 0.05 were accepted as significant.

## Results

### Structure of New Diphenylethane, 2′,3,5-Trihydroxy-4-Methoxybibenzyl (D1)

**FIGURE 1:**
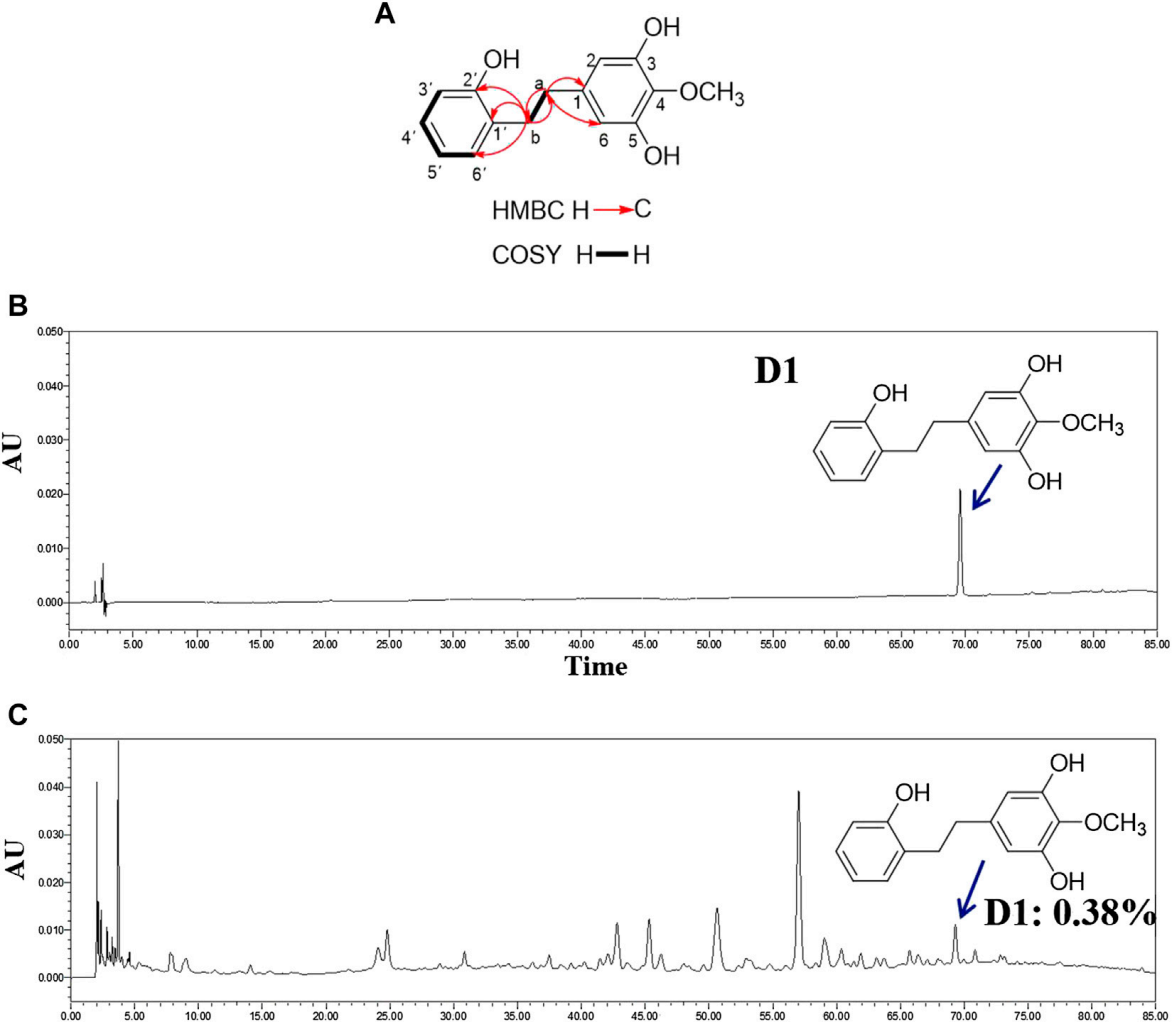
Structure and content of D1. **(A)**: Key ^1^H-^1^H COSY and HMBC correlations of D1. **(B)**: HPLC chromatogram of D1. **(C)**: HPLC chromatogram of extracts of stems and leaves of **(D)** *. oppositifolia* L. Waters Alliance 2,695 separations module equipped with Empower software hyphened with quaternary pumps, an automatic injector, a Waters 2,998 photodiode array (PDA) detector at 190–800 nm, and a 250 mm × 4.6 mm × 5 μm Platisil ODS C18 column was used for the separation. HPLC analysis conditions: Methanol **(A)**-Formic acid 0.1% **(B)**, 0–80 min (10–60% **(A)**); flow velocity: 1 ml/min, 15 μL injection; wavelength of detection: 260 nm; column temperatu: 30°C (Zeng et al., 2018).

D1 was isolated in the form of a brown oil. The molecular formula was determined to be C_15_H_16_O_4_ (eight degrees of unsaturation) based on HR-ESI-MS (*m/z* 261.1121 [M + H]^+^, calculated for 261.1121), which was consistent with the ^1^H and ^13^C NMR data (Table 1). The UV spectrum of D1 had aromatic absorptions at 203 and 275 nm. The IR spectrum displayed characteristic absorptions attributable to hydroxyl groups (3,363 cm^−1^) and aromatic rings (1,674 and 1,592 cm^−1^). The ^1^H NMR and HSQC spectra of D1 indicated a 1,2-disubstituted benzene ring [*δ*_H_ 6.99 (1H, d, *J* = 7.55 Hz, H-6′), 6.97 (1H, d, *J* = 7.85 Hz, H-4′), 6.74 (1H, d, *J* = 7.75 Hz, H-3′), and 6.71 (1H, d, *J* = 7.45 Hz, H-5′)], a 1,3,4,5-tetrasubstituted benzene ring [*δ*_H_ 6.22 (2H, s, H-2, 6)], a methoxy group [*δ*_H_ 3.75 (3H, s, OCH_3_-4)], and two methylene groups [*δ*_H_ 2.66 (2H, m, CH_2_-a) and 2.80 (2H, m, CH_2_-b)]. The ^13^C NMR and DEPT spectra of D1 indicated 15 carbons, including six sp2 methine groups [*δ*_C_ 131.1 (C-6′), 127.9 (C-4′), 120.4 (C-5′), 115.8 (C-3′), and 108.7 (C-2,6)], four oxyaryl carbons [156.3 (C-2′), 151.3 (C-3,5) and 134.8 (C-4)], an oxymethyl group [*δ*_C_ 60.8 (4-OCH_3_)], and two sp^3^ methylene groups [*δ*_C_ 37.1 (C-a) and 33.7 (C-b)]. These spectroscopic features suggested that the spectra of D1 was very similar to those of 3,5-dihydroxy-4-methoxybibenzyl (Yang et al., 2014) with an extra hydroxyl group at C-2′. Therefore, the structure of D1 was identified to be 2′,3,5-trihydroxy-4-methoxybibenzyl (Figure 1A). ^1^H and ^13^C NMR spectroscopic data of compound D1 is shown in Table 1. The content of D1 was 0.38% (Figures 1B,C).

**TABLE 1:** ^1^H and ^13^C NMR spectroscopic data of compound D1.

Position	1 (CD_3_OD)	
	*δ*_C_ (125 MHz)	*δ*_H_ (*J* in Hz) (500 MHz)
1	140.0	
2	108.7	6.22 s
3	151.3	
4	134.8	
5	151.3	
6	108.7	6.22 s
1′	129.6	
2′	156.3	
3′	115.8	6.74 days, (7.75)
4′	127.9	6.97 days, (7.85)
5′	120.4	6.71 days, (7.45)
6′	131.1	6.99 days, (7.55)
CH_2_-a	37.1	2.66 m
CH_2_-b	33.7	2.80 m
OCH_3_-4	60.8	3.75 s

### D1 Reduces Viability, Migration, Energy Metabolism, and Induced Apoptosis in A549 Cells

**FIGURE 2:**
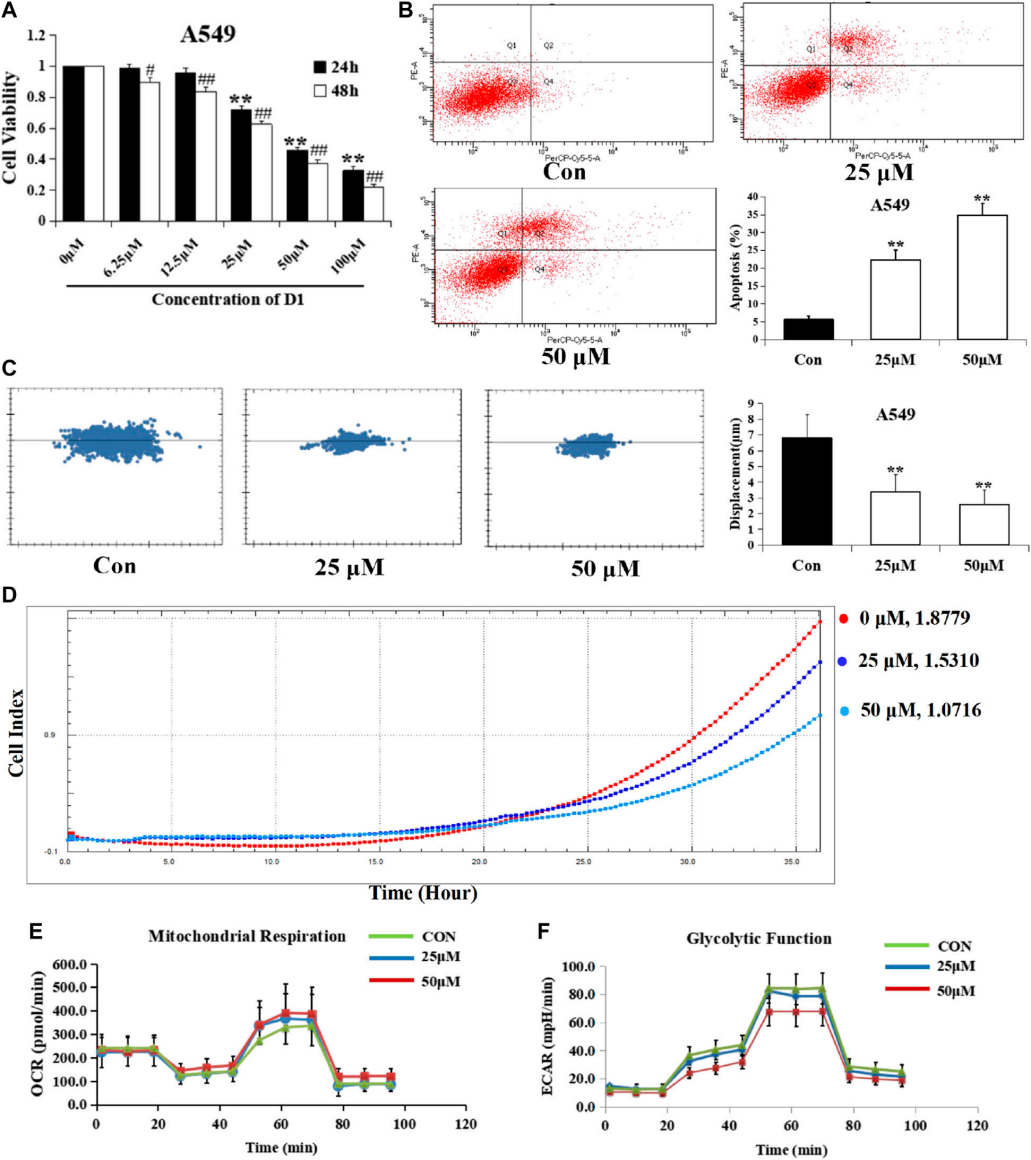
D1 Reduces Viability, Migration, Energy Metabolism, and Induced Apoptosis in A549 cells. **(A)**: Cell viability was measured by the MTT assay. A549 cells were treated with various concentrations of D1 or vehicle (Control) for 24 and 48 h, respectively. Data is shown as mean ± SD from three independent experiments, *p < 0.05, **p < 0.01 vs. Control (24 h); #*p* < 0.05, ##*p* < 0.01 vs. Control (48 h). **(B)**: A549 cells were treated with D1 (25 or 50 µM) or vehicle for 24 h. Apoptosis was analyzed by flow cytometry after 7-AAD/PE double staining. The percentage of apoptotic cells was presented as the mean ± SD of three independent experiments, **p < 0.01 vs. Control. **(C)**: A549 cells were plated in 96-well plates, the movement of A549 cells was monitored in real time by high-content imaging system followed by vehicle or D1 treatment. The displacement was normalized against the Harmony 4.8. **p < 0.01 vs. Control (total 24 h). **(D)**: A549 cells were plated in CIM-Plate16, xCELLigence system for real-time analysis of cell migration followed by vehicle or D1 treatment. **(E)**: OCA of A549 cells treated with D1. **(F)**: ECAR of A549 cells treated with D1.

The cytotoxicity of D1 was observed in the A549 cell line from the MTT assay. As shown in Figure 2A, D1 decreased A549 cell viabilities in both time and dose-dependent manner, with IC50 values of 38.9 µM and 29.2 µM after 24 and 48 h treatment, respectively. In addition, the high-content imaging system and xCELLigence system was performed to determine the effects of D1 on A549 cell migration. As shown in Figure 2D, after 48 h treatment, D1 significantly inhibited the migratory ability of A549 cells at the concentration of 25 or 50 µM. Likewise, a significant reduction of the cell displacement was observed in the D1 treatment groups (Figure 2C). Figure 2B shows that D1 induced apoptosis in a dose-dependent manner in the A549 cells. In addition, the results of Seahorse XF24 Analyzer showed that treatment with D1 transformed the extracellular acidification rate and oxygen consumption rate (Figures 2E,F).

### D1 Downregulates the STAT3 Target Gene Expression in A549 Cells

**FIGURE 3:**
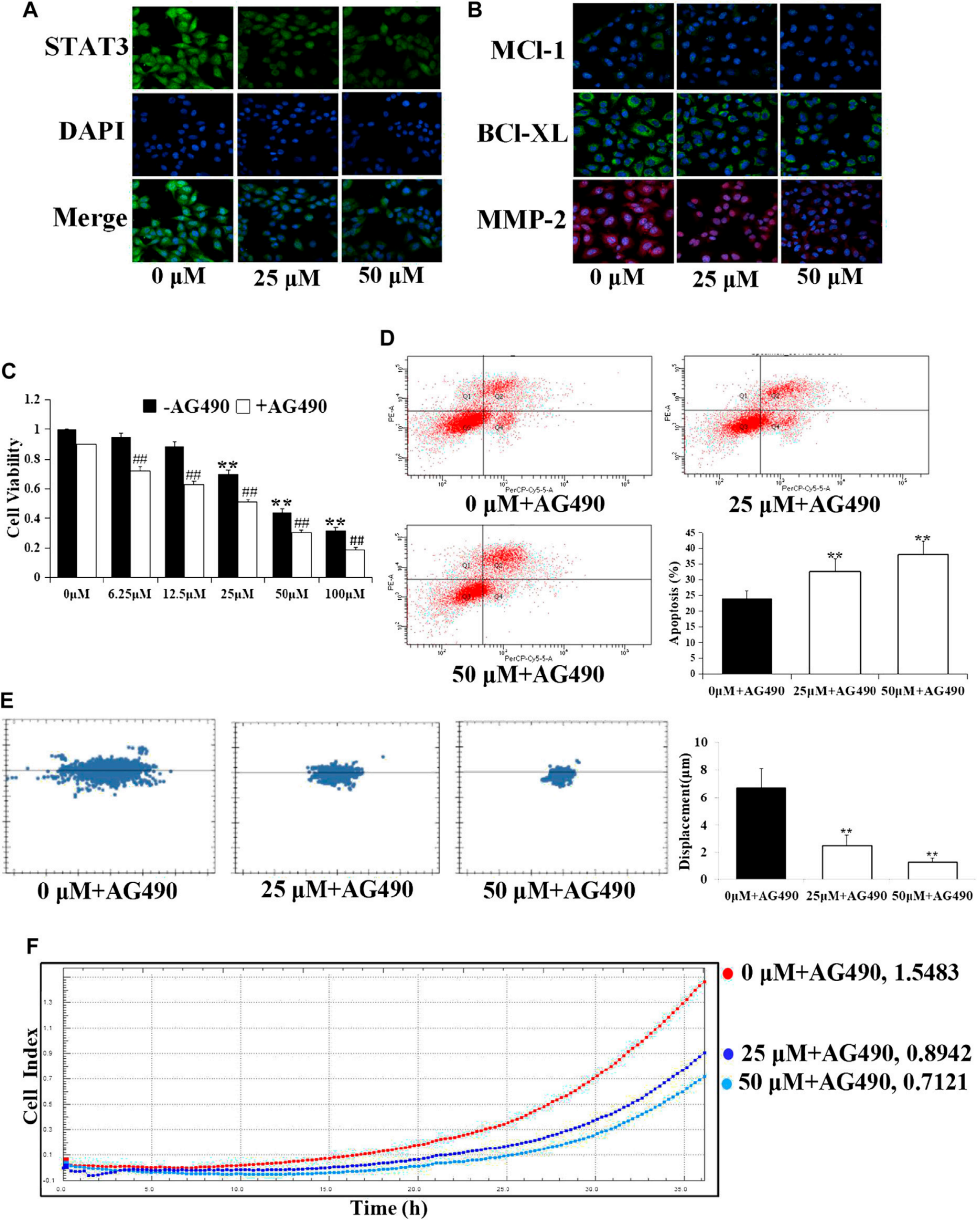
D1 downregulates the STAT3 target gene expression in A549 cells. A549 cells were treated with D1 (25 or 50 µM) for 24 h, cellular immunofluorescence by using antibodies specific to STAT3 **(A)**, Mcl-1, Bcl-xL and MMP-2 **(B)**. **(C)**: Cell viability was measured by the MTT assay. A549 cells were treated with D1 or AG490 for 24 h. Data were shown as mean ± SD from three independent experiments, **p < 0.01 vs. Control (- AG490); ## *p* < 0.01 vs. Control (+ AG490). **(D)**: A549 cells were treated with D1 and AG490 for 24 h. Apoptosis was analyzed by flow cytometry after 7-AAD/PE double staining. The percentage of apoptotic cells was presented as the mean ± SD of three independent experiments, **p < 0.01 vs. Control. **(E)**: A549 cells were plated in 96-well plates, the movement of A549 cells was monitored in real time by high-content imaging system followed by D1 and AG490 treatment. The displacement was normalized against the Harmony 4.8. **p < 0.01 vs. Control (24 h). **(F)**: A549 cells were plated in CIM-Plate16, xCELLigence system for real-time analysis of cell migration followed by D1 and AG490 treatment.

STAT3 dimerizes upon tyrosine phosphorylation at site 705, which leads to its nuclear translocation, and directly regulates a panel of tumor-promoting genes. As expected, the protein levels of STAT3 in nuclear fractions of A549 cells were significantly reduced by D1 in a dose-dependent manner (Figure 3A), moreover, we investigated, whether D1 affected the expression of STAT3 target genes including Mcl-1, Bcl-xL (involved in cell survival), and MMP-2 (involved in cell migration) (Su et al., 2017). As shown in Figure 3B, D1 dose-dependently decreased the levels of MMP-2, Mcl-1 and Bcl-xL. In addition, after nuclear translocation of STAT3 was inhibited by treatment with AG490 (a specific inhibitor of STAT3), D1 further reduced the viability, migration and induced apoptosis in A549 cells (Figures 3D–F).

### Estrogenic Activity of D1 Mediated by ER*β*

**FIGURE 4:**
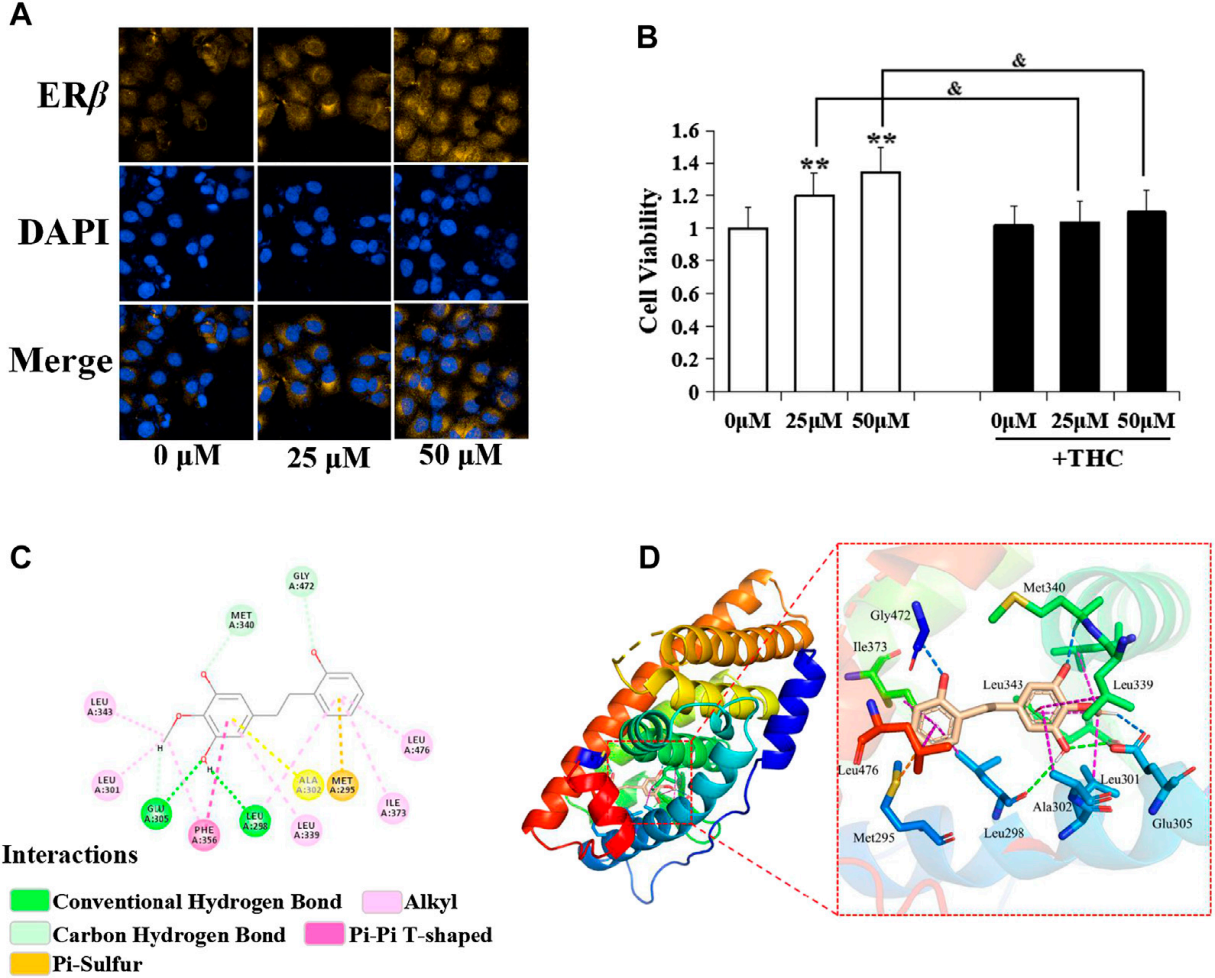
Estrogenic activity of D1 mediated by ER*β*. **(A)**: MCF-7 cells cultured in hormone-free medium (phenol red-free DMEM with 10% v/v charcoal-stripped FBS) were treated with D1 (25 or 50 µM) for 24 h, cellular immunofluorescence by using antibodies specific to ER*β*. **(B)**: Cell viability was measured by the MTT assay. MCF-7 cells cultured in hormone-free medium were treated with D1 or THC for 24 h. Data is shown as mean ± SD from three independent experiments, **p < 0.01 vs. Control (- THC); and *p* < 0.05 vs. same group (+ THC). **(C)**: The binding mode of D1 and ER*β* based on molecular simulation. 2D stereogram. **(D)**: 3D stereogram.

Figure 4B shows that D1 improved the proliferation rate of the MCF-7 cells, and THC (specific ER*β* antagonist, 1 µM) blocked the effect of D1 (25 or 50 µM) on the MCF-7 cell proliferation. Moreover, D1 (25 µM) upgraded the expression of ER*β* in MCF-7 cells (Figure 4A). To further investigate the molecular interaction between D1 and ER*β*, the structure of ER*β* (PDB: 1U3Q) and D1 was studied using the molecular simulations. As shown in Figures 4C,D, four H-bond and eight hydrophobic interactions occurred in D1 with the active pocket of ER*β*. These results suggested that D1 was a potential ER*β* activator. D1 had no effect on ER*α* (results not shown).

### D1 Inhibits Migration and Induced Apoptosis via ERβ-STAT3 Pathway in A549 Cells

**FIGURE 5:**
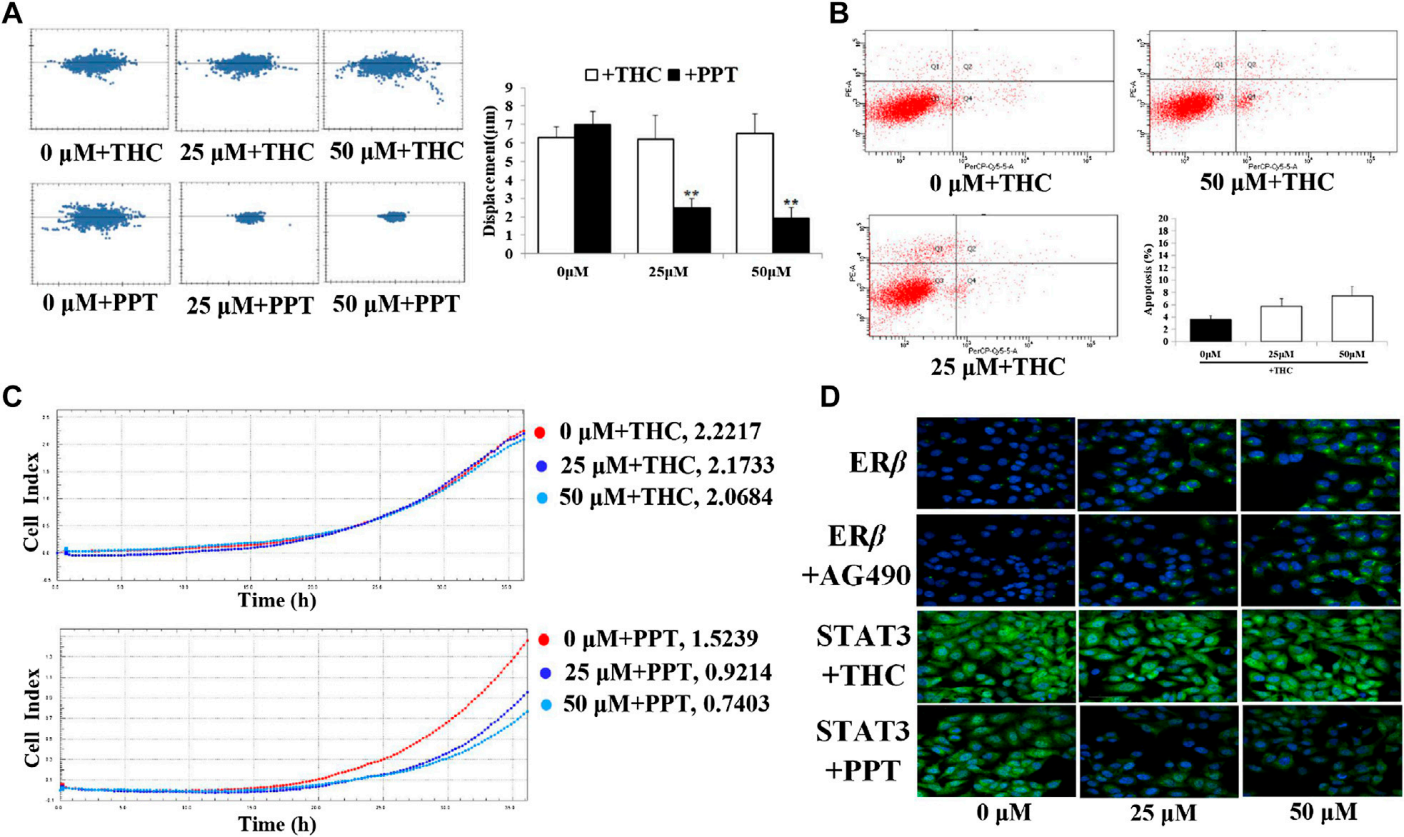
D1 inhibits migration and induced apoptosis via ER*β*-STAT3 pathway in A549 cells. **(A)**: A549 cells were plated in 96-well plates, and the movement of the cells was monitored in real time by high-content imaging system after D1 and THC or PPT treatment. The displacement was normalized against the Harmony 4.8. **p < 0.01 vs. Control (total 24 h). **(B)**: A549 cells were treated with D1 and THC for 24 h. Apoptosis was analyzed by flow cytometry after 7-AAD/PE double staining. Data was presented as the mean ± SD of three independent experiments. **(C)**: A549 cells were plated in CIM-Plate16, xCELLigence system for real-time analysis of cell migration followed by D1 and THC or PPT treatment. **(D)**: A549 cells were treated with D1 and AG490, THC or PPT for 24 h, cellular immunofluorescence by using antibodies specific to ERβ and STAT3.

Specific estrogen receptor β blocker (THC), specific estrogen receptor β agonist (PPT), and STAT3 inhibitor (AG490) were administered respectively 30 min before the treatment of D1 to evaluate whether the observed effects elicited by D1 were mediated via the ER*β*-STAT3 pathway. Results of the immunofluorescence experiments revealed that THC increased the nuclear translocation of STAT3, but AG490 was unable to block the expression of ER*β* (Figure 5A). Moreover, PPT decreased the nuclear translocation of STAT3. In addition, the downregulation of migration and the upregulation of apoptosis by D1 in A549 cells was found to be efficiently blocked by THC. Meanwhile, D1 promoted the migration and induced apoptosis in A549 cells, which was further activated by PPT (Figures 5B–D).

## Discussion

Lung cancer has become the type of cancer with the highest incidence among all the other types cancers in the world, and it appears to affect more among the young people (Xiao et al., 2020). The existing treatment methods have disadvantages such as poor selectivity, high toxic side-effects and a rapid development of drug resistance (Zhang et al., 2017). Therefore, the exploration of new drugs and new methods for the treatment of lung cancer requires an urgent attention. A large number of basic research and clinical practice have shown that natural products can play an important role in the tumour prevention and rehabilitation of cancer patients (Kim et al., 2020; Liu et al., 2020; Yeh et al., 2020). Their reliable source and high safety are the features, which make them the main source of new drug development. Currently, the anti-cancer preparations derived from the plant source account for approximately 32% of the total anti-cancer drugs, such as colchicine, vinblastine, paclitaxel are all isolated from natural products (Fatima et al., 2019; Zhou et al., 2019; Modi et al., 2020). In the present study, a new diphenylethane compound (D1) was obtained for the first time, its effects on A549 cells were studied, and its possible mechanism of action was explored. We aimed to unearth compounds with effective anti-NSCLC efficacy from stems and leaves of *Dioscorea oppositifolia* L., just like the discovery of artemisinin from a traditional anti-malarial Chinese medicinal herb *Artemisia annua*. We hope to screen out a bioactive components-enriched fraction from the studied stems and leaves of *Dioscorea oppositifolia* L. for developing an anti-NSCLC agent, because it has been well accepted that natural products with multi-components and multi-targets natures have advantages in managing the complex disease such as cancer (Su et al., 2017).

STAT3 is an important member of the signal transduction and activation protein (STAT) family (Kadye et al., 2020). It is known to participate in the regulation of the transcription of multiple target genes related to the tumour occurrence and development, and cause abnormal expression of proteins related to proliferation, differentiation, cycle and apoptosis, thereby causing tumour cell anti-apoptosis, promoting cell proliferation and migration (Sen et al., 2020). In the present study, it was found that D1 reduced the viability, migration, energy metabolism and induced the apoptosis in A549 cells. It is known that, upon phosphorylation, STAT3 dimerizes and translocates into the nucleus to regulate the transcription of its target genes. Therefore, we speculated that the inhibition of the STAT3 phosphorylation could result in the reduced STAT3 nuclear localization and decreased expression of genes that are transcriptionally upregulated by STAT3 (Su et al., 2017). Indeed, D1 treatment reduced the nuclear localization of STAT3 and lowered the expression of STAT3 targeted Bcl-xL, MMP-2 and Mcl-1 that are involved in A549 cell proliferation, survival and mobility.

Phytoestrogens are a class of natural plant compounds with weak estrogen effects. Its main mechanism is to exert estrogen-like or anti-estrogen effects by binding to estrogen receptors (ERα, ER*β*). They have two-way regulatory functions and are effective on the prevention and treatment of hormone-related diseases (Francesco and Petropoulos, 2019; Najafi, 2019). Using the estrogen-deprived MCF-7 cell model is a common method to test whether drugs have the estrogenic activity *in vitro*. When drugs with estrogen-like activity are co-cultured with the de-estrogen MCF-7 cells, the de-estrogen MCF-7 cells proliferate significantly, and the interference of exogenous estrogen is excluded (Kewen et al., 2016; Zeng et al., 2018). In this study, we found that D1 exhibited an estrogenic activity and promoted the proliferation of estrogen-deprived MCF-7 cells, and that the pro-proliferation effect could be blockedby THC (a ER*β* specific antagonist) (Watanabe et al., 2004). Cellular immunofluorescence results showed that D1 increased the expression of ER*β*, while the molecular docking results showed that D1 binds to the ER*β* significantly, indicating that the estrogen-like activity of D1 is mediated by ER*β*. SPR assay will be used to explore D1 targeting on ERβ in subsequent research.

Studies have shown that the survival time of premenopausal female NSCLC patients was significantly shorter than that of postmenopausal female patients, and that the tumour was more aggressive (Moore et al., 2003; Hsu et al., 2015). The local estrogen content of cancer tissues in postmenopausal female patients was significantly lower than that of men (Niikawa et al., 2008). Therefore, it is speculated that endogenous hormones of the female patients can promote the occurrence and development of NSCLC. D1 has estrogen-like activity, and we speculate that it can compete with the endogenous estrogen to bind to the estrogen receptor, thereby preventing the further development of NSCLC. We found that D1 can significantly reduce the viability and migration of A549 cells, and that this effect was blocked by THC (a ERβ specific antagonist) and be further activated by PPT (a ERβ specific agonist) (Brenna et al., 2016). This indicated that D1 exerts its anti-NSCLC effect through the ER*β* pathway. Moreover, our results showed that inhibiting the nuclear translocation of STAT3 did not interfere with the binding of D1 to the ER*β* receptor. On the contrary, after antagonizing ER*β*, the nuclear translocation of STAT3 increased, indicating that STAT3 could be a downstream signalling molecule of ER*β*, and that D1 could exhibit its anti-NSCLC function through the ER*β*-STAT3 pathway.

## Conclusion

In the present study, we identified a new diphenylethane (D1) from the stems and leaves of *D. oppositifolia* L., which reduced the cell viability, migration, energy metabolism and induced cell apoptosis in A549 cells. Mechanistic studies showed that D1 reduced the STAT3 nuclear localization and downregulated the expression STAT3 target genes. It was observed that D1 exhibited estrogenic activities mediated by ER*β*, and the antagonism of ER*β* decreased the cytotoxic effect of D1 in A549 cells. Additionally, inhibiting the nuclear translocation of STAT3 did not interfere with the binding of D1 and ER*β*, but after antagonizing ER*β*, the nuclear translocation of STAT3 increased, thereby demonstrating that STAT3 was the downstream signaling molecule of ER*β*. Our results indicate that D1 exhibited an *in vitro* anti-NSCLC effect, which could be partially due to the modulation of the ER*β-*STAT3 signaling pathway. We speculate the potential of D1 to be developed as a modern alternative and/or complimentary agent in the management of NSCLC, and also improve the comprehensive utilization rate of *D. oppositifolia* L.

## Data Availability

The original contributions presented in the study are included in the article/Supplementary Material, further inquiries can be directed to the corresponding authors.
